# LncRNA NEAT1 facilitates glioma progression via stabilizing PGK1

**DOI:** 10.1186/s12967-022-03273-2

**Published:** 2022-02-05

**Authors:** Jingshan Liang, Changtao Liu, Dezhi Xu, Kang Xie, Aimin Li

**Affiliations:** Lianyungang Clinical College of Nanjing Medical University/The First People’s Hospital of Lianyungang, No. 182, Tongguan Road, Lianyungang, 222000 China

**Keywords:** Glioma, NEAT1, PGK1, lncRNA, Glycolysis

## Abstract

**Background:**

Long noncoding RNA NEAT1 has been implicated in glioma progression. However, the effect of NEAT1 on glycolysis of glioma cell and the potential mechanism remain unclear.

**Methods:**

In vitro experiments, including CCK-8, colony formation, ECAR, and lactate detection assays were performed to evaluate the effect of NEAT1 on proliferation and glycolysis of glioma cell. RNA pulldown and RIP assays were performed to identify the interaction between NEAT1 and PGK1. Truncated mutation of NEAT1 and PGK1 was used to confirm the specific interactive domains between NEAT1 and PGK1. Animal studies were performed to analyze the effect of NEAT1/PGK1 on glioma progression.

**Results:**

NEAT1 knockdown significantly suppressed the proliferation and glycolysis of glioma cells. NEAT1 could specifically interact with PGK1, which promotes PGK1 stability. Hairpin A of NEAT1 is essential for interaction with M1 domain of PGK1. Depletion of NEAT1 markedly inhibited tumor growth in mice, while PGK1 could reverse this effect. Higher expression of NEAT1 was associated with poor overall survival of GBM patients.

**Conclusions:**

NEAT1 over expression promotes glioma progression through stabilizing PGK1. NEAT1/PGK1 axis is a candidate therapeutic target for glioma treatment.

## Introduction

Glioma is one of the most malignant and prevalent tumor in adults [1, 2]. Despite of the aggressive therapy including maximal surgical resection together with radio- and chemo- therapy, the survival time of glioblastoma patients is still less than 14 months [3, 4]. Therefore, understanding the underlying mechanism of glioma progression is essential for improving patients’ prognosis.

Long non-coding RNAs (lncRNAs) are a class of RNAs that consists of more than 200 nucleotides and lacks the ability of coding proteins [5, 6]. Nuclear paraspeckle assembly transcript 1 (NEAT1) has been reported to be an important lncRNA participating in progression of a lot of tumors, including glioma [7–10]. NEAT1 was shown closely associated with glioma patients’ prognosis [11]. And, NEAT1 levels were characteristically overexpressed in glioma cell lines [12]. However, little is known about the mechanism of NEAT1 regulating glioma development. By now, most researches of NEAT1 focused on its role of sponging miRNAs, like miR-107 [13] and miR-139-5p [14]. Qun Chen et al. [10] identified that NEAT1 was critical for glioma progression by increasing β-catenin nuclear transport and H3K27 trimethylation. However, these results did not revealed the mechanism of NEAT1 on regulating glycolysis of glioma cells.

In this study, we found that NEAT1 was overexpressed in glioma. NEAT1 knockdown significantly suppressed proliferation and glycolysis of glioma cell. In addition, we identified a new NEAT1 interacted protein: PGK1. NEAT1 overexpression is essential for PGK1 stability in glioma cells, thus promoting glycolysis of glioma cells. Moreover, we identified the direct interaction between hairpin A of NEAT1 and M1 domain of PGK1. Lastly, NEAT1 expression was positively correlated with PGK1 in glioma samples, indicating NEAT1 and PGK1 are potential biomarkers for glioma.

## Method and materials

### Clinical samples

Five tumor adjacent tissues and twenty GBM samples were collected from The department of Neurosurgery, Lianyungang Clinical College of Nanjing Medical University. Samples were surgically resected and frozen immediately in liquid nitrogen for further study. The use of clinical samples was approved by Lianyungang Clinical College of Nanjing Medical University.

### Cell culture

The human glioma cell lines (U251 and T98G) were purchased from ATCC. Cells were maintained in DMEM (Gibco, USA) supplemented with 10% FBS (Fetal Bovine Serum) (Hyclone, USA), 100 μg/ml penicillin, and 100 μg/ml streptomycin in 37 ℃. Cells were thawed fresh every 2 months.

### Cell transfection

Polymerase chain reaction (PCR)-amplified full length or truncated human PGK1 was cloned into pcDNA3.1/hygro( +)-Flag. And the authenticity of all the constructs was confirmed by DNA sequencing. PGK1 CDS was amplified using primers described below:

**Table:** 

	Forward	Reversed
Full length PGK1	AATGCGGCCGCATGTCGCTTTCTAACAAG	GCCTCTAGACTAAATATTGCTGAGAGCATC
PGK1 M1	AATGCGGCCGCATGTCGCTTTCTAACAAG	GCCTCTAGATACACAGTCCTTCAAGAACAG
PGK1 M2	AATGCGGCCGCGGCCCAGAAGTGGAGAAA	GCCTCTAGAGGCCTTTGCAAAGTAGTTCAG
PGK1 M3	AATGCGGCCGCTTGGAGAGCCCAGAGCGA	GCCTCTAGATTGGCCAGTCTTGGCATTCTC
PGK1 M4	AATGCGGCCGCGCCACTGTGGCTTCTGGC	GCCTCTAGACTAAATATTGCTGAGAGCATC
PGK1 ΔM4	AATGCGGCCGCATGTCGCTTTCTAACAAG	GCCTCTAGATTGGCCAGTCTTGGCATTCTC

LipofectamineTM 2000 reagent was used for plasmids transfection. Lentivirus carrying NEAT1 shRNA (the target sequence of NEAT1-shRNA is GCGCAAGTTAGCCACAAAT) was purchased from Ribobio (China) and transfected into GBM cells according to the manufacturer’s instruction.

### RNA extraction and qRT-PCR analysis

Total RNA from U251 and T98G cells was extracted using TRIzol reagent (Invitrogen, CA) as previously described [15]. qRT-PCR was performed using SYBR Green Premix Ex Taq (TaKaRa) according to the manufacturer’s recommendations. The sequence information of NEAT1 primers used in this study was shown below.

Forward: CCAGTTTTCCGAGAACCAAA.

Reversed: ATGCTGATCTGCTGCGTATG.

### Protein extraction and western blot analysis

Protein samples preparation and western blot analysis were performed as previously described [15]. Briefly, glioma cells were lysed using RIPA lysis buffer supplemented with protease inhibitors. Next, cell lysates were centrifugated at 12,000*g* for 15 min at 4 ℃. The protein samples were separated by 10% SDS-PAGE and transferred to nitrocellulose filter membranes (Millipore). 5% nonfat milk was used to block the membranes for 2 h. After washing for three times using PBS/Tween-20, the membranes were incubated with the primary antibodies. Antibodies against CDK2 (#18048, Cell Signaling Technology), CDK4 (#12790, Cell Signaling Technology), CDK6 (#13331, Cell Signaling Technology), PGK1 (#68540, Cell Signaling Technology), Flag (F9291, Sigmaaldrich), HA (#3724, Cell Signaling Technology), and GAPDH (#5174, Cell Signaling Technology) were used in this study. SuperSignal West Femto Maximum Sensitivity Substrate (Thermo) was used to visualize subsequent. The western blotting results was quantified using Image J software.

### CCK-8 assays

CCK-8 assays were performed to evaluate cell growth. Briefly, 2000 transfected U251 and T98G cells were seeded into a 96-well plates. CCK-8 reagent was added at indicated time and incubated for 1 h at 37 ℃. The absorbance was detected at 450 nm.

### Colony formation assays

Colony formation assays were performed to evaluate cell growth. Briefly, 400 transfected U251 and T98G cells were seeded into a 6-well plate. And cells were maintained until colonies formed. Colonies were then fixed by paraformaldehyde for 30 min and stained using crystal violet. The colonies were then calculated for further analysis.

### Extracellular acidification rate analysis

The XF Glycolysis Stress Test kit was used for the ECAR assay. Briefly, 1 × 10^4^ transfected U251 and T98G cells were seeded into the Seahorse SF 96 cell culture microplates. 10 mM glucose, 1 um oligomycin, and 75 mM 2-DG were added to the cell medium successively at indicated time. Next, Seahorse SF-96 Wave software was used to analyze the results. ECAR was presented in mpH/min.

### Measurement of intracellular Lactate levels

The accumulation of lactate in transfected glioma cells was measured using lactate assay kit II (Sigma Aldrich, MAK065). Briefly, glioma cells were homogenized and centrifuged at 13,000*g* for 10 min. The supernatant was collected and deproteinized using a 10 kDa MWCO spin filter to remove lactate dehydrogenase. Reaction Mixes were prepared according to manufacture. Add 50 μl mix to a 96-well plate and incubated the reaction for 30 min at room temperature. Measure the absorbance at 450 nm.

### RNA pulldown assay

Biotin-labeled RNA NEAT1 was transcribed in vitro using the T7 High Yield RNA Transcription Kit (Vazyme) and Pierce™ RNA 3’ End Desthiobiotinylation Kit (Thermo Fisher). Next, Pierce™ Magnetic RNA–protein Pull-Down Kit (Thermo Fisher) was used to obtain the RNA–protein binding mixture. Mass spectrometry was performed to identify target proteins.

### RNA immunoprecipitation (RIP) assay

Magna RIP RNA Binding Protein Immunoprecipitation Kit (Millipore) was used for RIP assays. Briefly, whole cell lysis was extracted from 1 × 10^5^ U251 and T98G cells. The protease inhibitor cocktail and RNase inhibitor were added into the cell lysis for 5 min. Meanwhile, magnetic beads were incubated with IgG or Flag antibodies for half an hour at room temperature. After centrifugating at 1000*g* for 10 min, the supernatant of cell lysis was incubated with coated beads and incubated at 4 ℃ overnight. At last, the purified RNA was quantified by qRT-PCR.

### Animal study

The animal manipulations were approved by Animal Core Facility of Nanjing Medical University. 1 × 10^6^ U251 cells, which were transfected with luciferase reporter, were suspended in 10 ul of DMEM, and intracranially injected into nude mice (female, 6-week-old). The tumor growth was monitored by bioluminescence imaging system at indicated time. At the end, mice were euthanatized by cervical dislocation under anesthesia, and the tumors from brains were fixed and embed using paraffin for further IHC analysis.

### Immunohistochemistry analysis

Paraffin-embedded clinical human and mice samples were sliced into 4 mm slides followed by dewaxing and rehydration. Antibodies against ki-67 (#9027, Cell Signaling Technology) and PGK1 (ab233135, abcam) were incubated with tissues overnight at 4 ℃. The protein expression was detected using DAB (Gene Tech). The IHC score was judged by two independent pathologists. The following proportion scores were assigned: 0, 1, 2, 3, 4, and 5 if 0%, 0%–1%, 2%–10%, 11%–30%, 31%–70%, and 71%–100% of the tumor cells exhibited positive staining, respectively. Also, the staining intensity was rated on a scale of 0 to 3: 0, negative; 1, weak; 2, moderate; and 3, strong. A total score was obtained by adding the proportion score and intensity score.

### Statistic analysis

Student t test or one-way ANOVA was performed to evaluate differences between two groups or between more than two groups, respectively. Unless stated otherwise, experiments were performed for three time and data represent the mean ± SD. Overall survival rates were calculated by the Kaplan–Meier method with the log-rank test applied for comparison. *p* < 0.05 was considered as statistically significant. All statistical analyses were performed using GraphPad Prism 8 software.

## Results

### NEAT1 promotes glioma cell proliferation and glycolysis

**Fig. 1:**
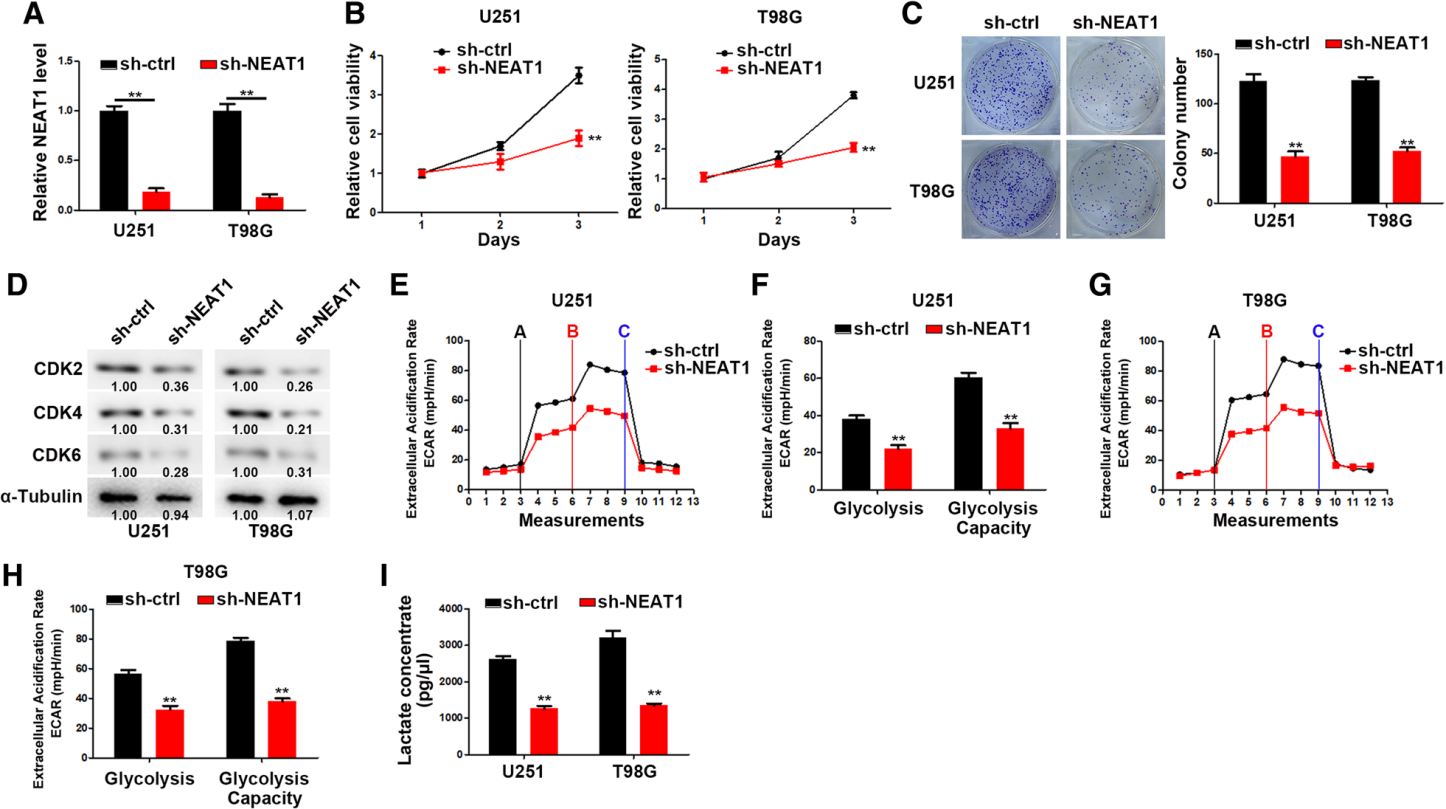
NEAT1 promotes cell proliferation and glycolysis. **A** Relative NEAT1 expression levels in U251 and T98G cells transfected with sh-ctrl or sh-NEAT1.** B** CCK-8 analysis of U251 and T98G cells transfected with sh-ctrl or sh-NEAT1.** C** Colony formation assays of U251 and T98G cells transfected with sh-ctrl or sh-NEAT1.** D** Western blot analysis of CDK2, CDK4, and CDK6 in U251 and T98G cells transfected with sh-ctrl or sh-NEAT1. **E**, **F** ECAR was measured through the Glycolysis Stress in U251 cells transfected with sh-ctrl or sh-NEAT1. **G**, **H** ECAR was measured through the Glycolysis Stress in T98G cells transfected with sh-ctrl or sh-NEAT1.** I** Lactate concentration was assessed in U251 and T98G cells transfected with sh-ctrl or sh-NEAT1. *p < 0.05, **p < 0.01. Error bars indicate mean ± SD of triple independent experiments

We firstly generated NEAT1 knockdown GBM cells by sh-NEAT1 transfection (Fig. 1A). CCK-8 and colony formation assays were performed to analyze the proliferation of GBM cells. As shown in Fig. 1B, C, NEAT1 knockdown significantly inhibited the GBM cell growth. In addition, the expression of cell cycle regulatory proteins were detected and the results indicated that NEAT1 knockdown strikingly suppressed CDK2, CDK4 and CDK6 expression (Fig. 1D). Furthermore, glycolysis rate and glycolytic capacity were significantly reduced followed by NEAT1 knockdown (Fig. 1E–H). Moreover, NEAT1 knockdown markedly decreased the intracellular metabolites of glycolysis in GBM cells (Fig. 1I).

Taken together, these data suggested that NEAT1 in functionally important for GBM cell growth and metabolic reprogramming.

### NEAT1 directly interacts with PGK1

**Fig. 2:**
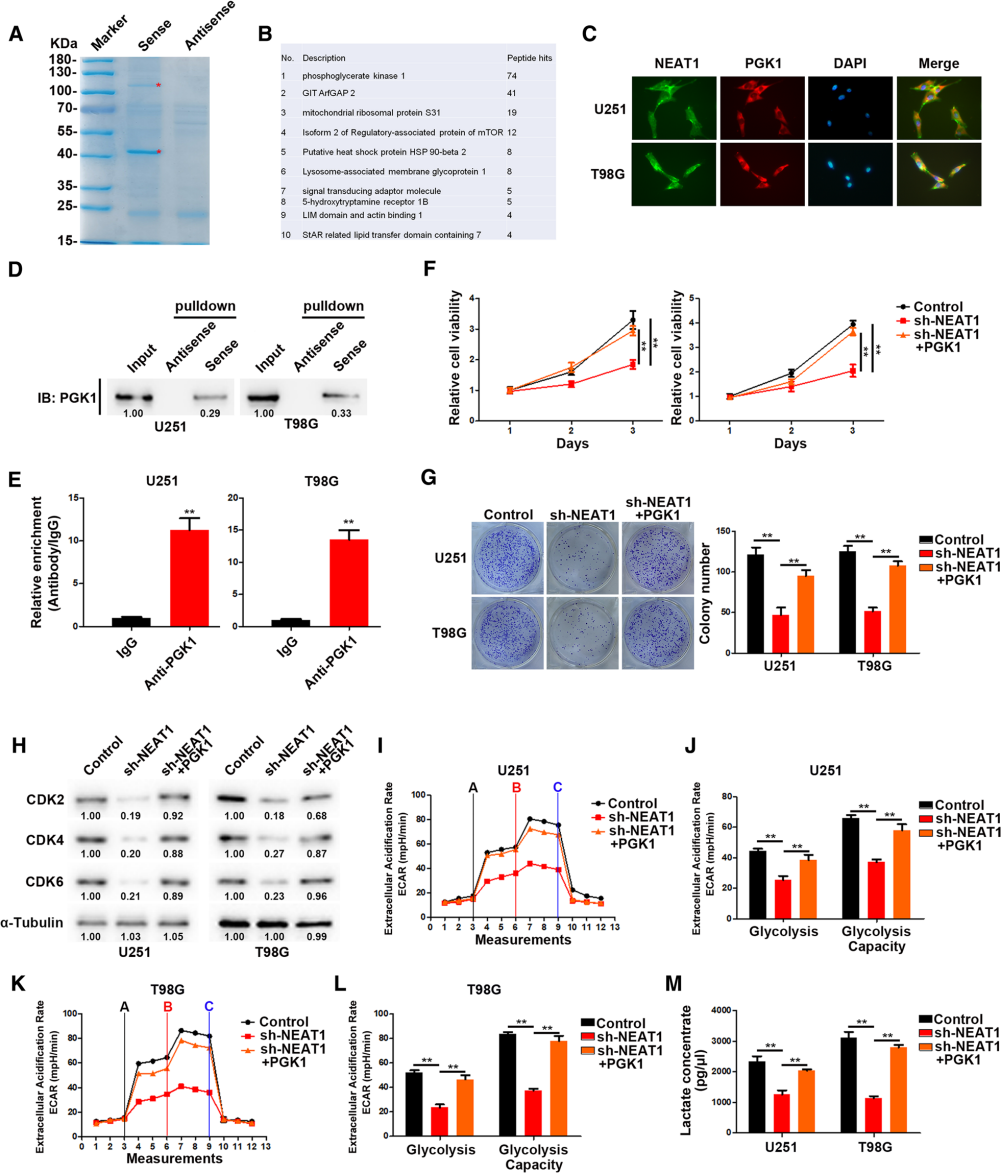
NEAT1 directly interacts with PGK1. **A** Coomassie brilliant blue staining of NEAT1 pulldown. Asterisks show different bands between the sense and antisense lanes. **B** List of the top 10 differentially expressed proteins identified by mass spectrometry. **C** Confocal images showing subcellular localization of NEAT1 and PGK1 in U251 and T98G cells. **D** NEAT1 pull-down followed by western blot exhibited the binding of NEAT1 to PGK1. **E** RIP assay showed the binding of NEAT1 to PGK1. **F** CCK-8 analysis of U251 and T98G cells transfected with sh-NEAT1 or co-transfected with sh-NEAT1 and PGK1. **G** Colony formation assays of U251 and T98G cells transfected with sh-NEAT1 or co-transfected with sh-NEAT1 and PGK1. **H** Western blot analysis of CDK2, CDK4, and CDK6 in U251 and T98G cells transfected with sh-NEAT1 or co-transfected with sh-NEAT1 and PGK1. **I**, **J** ECAR was measured through the Glycolysis Stress in U251 cells transfected with sh-NEAT1 or co-transfected with sh-NEAT1 and PGK1. **K**, **L** ECAR was measured through the Glycolysis Stress in T98G cells transfected with sh-NEAT1 or co-transfected with sh-NEAT1 and PGK1. **M** Lactate concentration was assessed in U251 and T98G cells transfected with sh-ctrl or sh-NEAT1. *p < 0.05, **p < 0.01. Error bars indicate mean ± SD of triple independent experiments

To dissect the potential molecular mechanism of NEAT1 regulating proliferation and glycolysis of glioma cells, we designed a biotin-conjunct probe followed by RNA pulldown assay and protein mass spectrometry analysis. As shown in Fig. 2A, B, NEAT1 could specifically interact with PGK1. Confocal microscopy confirmed the co-localization of NEAT1 with PGK1 in the cytoplasm of U251 and T98G cells (Fig. 2C). We verified the interaction between NEAT1 and PGK1 by RNA pulldown followed western blot analysis. The results showed that biotin-labeled NEAT1 could pull down abundant PGK1 compared with anti-sense (Fig. 2D). In addition, RIP assays were performed and the results indicated that anti-PGK1 antibody could pull down plentiful NEAT1 compared to IgG control (Fig. 2E). These results confirmed the interaction between NEAT1 and PGK1. Moreover, we overexpressed PGK1 in NEAT1-depleted GBM cells. As expect, PGK1 overexpression could reverse the suppressed cell proliferation and glycolysis caused by NEAT1 knockdown (Fig. 2G–M).

Taken together, our results indicated that NEAT1 promoted cell proliferation and glycolysis by coordinating with PGK1.

### NEAT1 inhibits PGK1 degradation via the ubiquitin–proteasome pathway

**Fig. 3:**
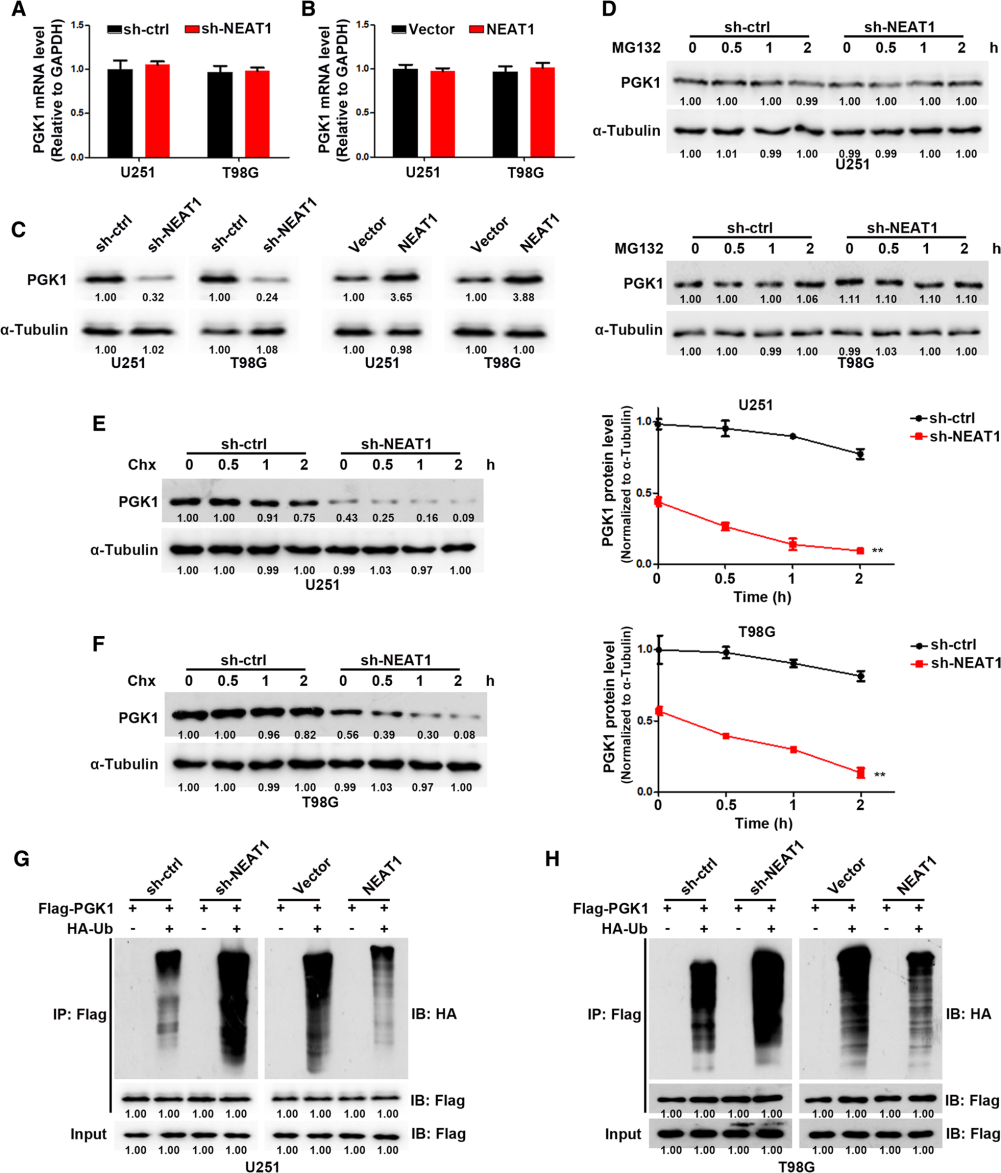
NEAT1 inhibits PGK1 degradation via the ubiquitin–proteasome pathway. **A** Relative PGK1 mRNA levels in U251 cells transfected with sh-ctrl or sh-NEAT1. **B** Relative PGK1 mRNA levels in T98G cells transfected with sh-ctrl or sh-NEAT1. **C** Western blot analysis of PGK1 in U251 and T98G cells transfected with sh-NEAT1 or NEAT1. **D** Western blot analysis of PGK1 in transfected U251 and T98G cells treated with MG132 for the indicated time. **E** Western blot analysis of PGK1 in transfected U251 cells treated with Chx for the indicated time (left). The quantification of PGK1 degradation rate by gray scale analysis (right). **F** Western blot analysis of PGK1 in transfected T98G cells treated with Chx for the indicated time (left). The quantification of PGK1 degradation rate by gray scale analysis (right). **G** Ubiquitinated PGK1 detected by immunoprecipitation with anti-Flag antibody in transfected U251 cells. **H** Ubiquitinated PGK1 detected by immunoprecipitation with anti-Flag antibody in transfected T98G cells. *p < 0.05, **p < 0.01. Error bars indicate mean ± SD of triple independent experiments

Next, we examined the expression of PGK1 in GBM cells with or without NEAT1 knockdown. As shown in Fig. 3A, B, NEAT1 had no effect on PGK1 mRNA levels in GBM cells. However, in NEAT1-depleted GBM cells, PGK1 protein levels were dramatically reduced (Fig. 3C). Therefore, we proposed that NEAT1 could regulate PGK1 level via post-translational modification. We then inhibited protein de novo synthesis or proteasomal degradation in GBM cells with cycloheximide (CHX) or MG132, respectively. After MG132 treatment, PGK1 protein levels remained stable in NEAT1-depleted or control GBM cells, indicating that NEAT1 did not affect PGK1 protein synthesis (Fig. 3D). However, as shown in Fig. 3E, F, the expression of PGK1 protein rapidly decreased in NEAT1-depleted GBM cells in the presence of CHX, while PGK1 protein expression remained high in control GBM cells, indicating that NEAT1 knockdown significantly decreased the protein stability and half-life of PGK1. Previous studies showed that the degradation of proteins is largely duo to the ubiquitin–proteasome pathway [16]. To investigate whether NEAT1 regulates the ubiquitination of PGK1, we co-expressed Flag-PGK1 and HA-ubiquitin in both NEAT1-depleted and control GBM cells. After IP PGK1 from GBM cells pretreated with MG132, we observed that, compared with control cells, PGK1 was heavily ubiquitinated in NEAT1-depleted cells; On the contrary, PGK1 overexpression induced decreased ubiquitination level in GBM cells (Fig. 3G, H). Together, these results indicated that NEAT1 increases the protein level of PGK1 by inhibiting ubiquitination-induced protein degradation.

### Hairpin A of NEAT1 interacts with M1 domain of PGK1 to promote cell proliferation and glycolysis

**Fig. 4:**
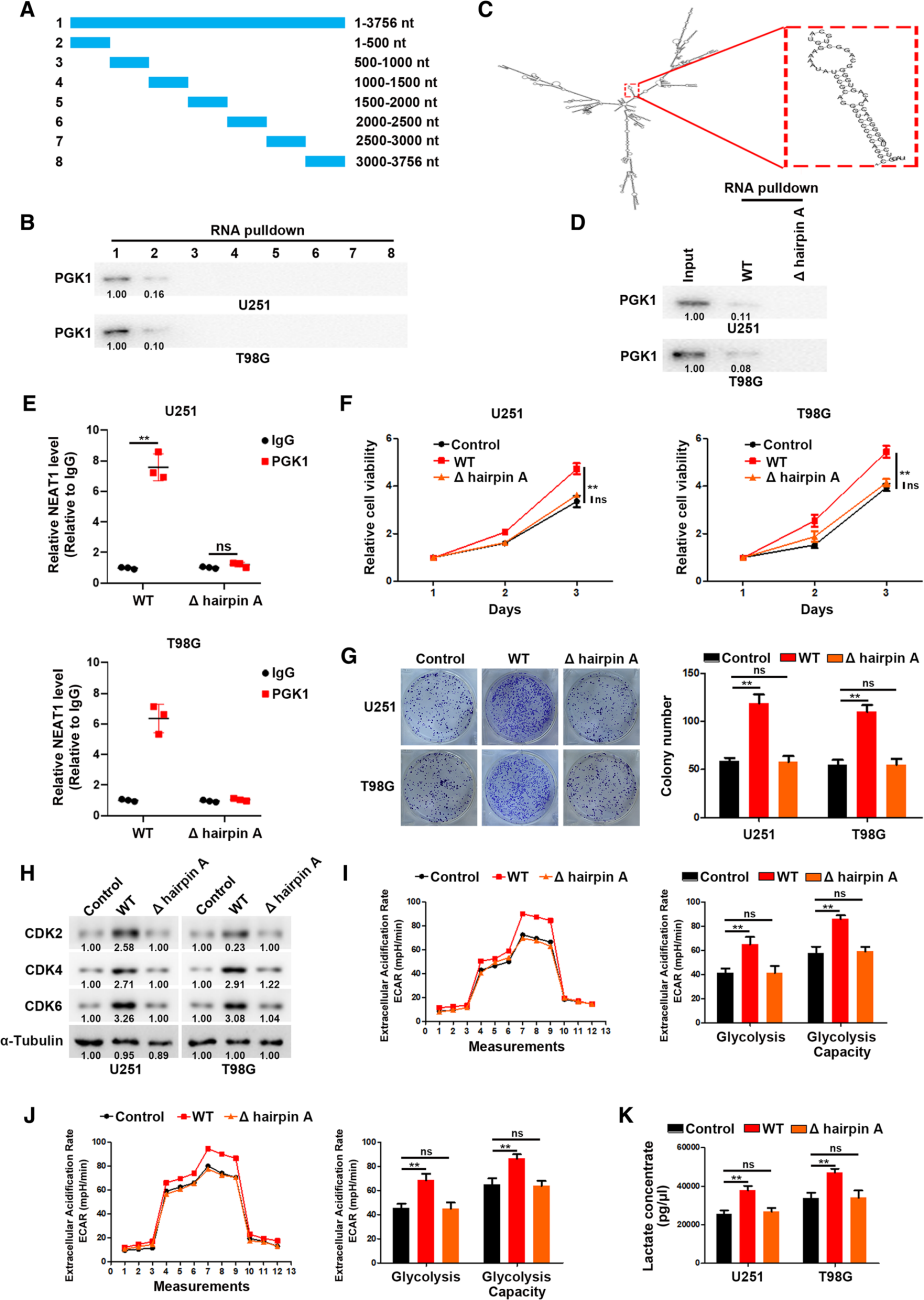
Hairpin A of NEAT1 interacts with PGK1. **A** A schema of truncated NEAT1. **B** RNA pull-down via sequential truncated NEAT1 fragments showed the binding region of NEAT1 with PGK1. **C** The structure of NEAT1 predicted by RNAfold indicates a stable stem-loop (hairpin A) structure within 1–500 nt. **D** The deletion of hairpin A abolished the binding of NEAT1 with PGK1. **E** RIP assays performed after the deletion of hairpin A in U251 and T98G cells. **F** CCK-8 analysis of U251 and T98G cells transfected with WT or hairpin A-deleted NEAT1. **G** Colony formation assays of U251 and T98G cells transfected with WT or hairpin A-deleted NEAT1. **H** Western blot analysis of CDK2, CDK4, and CDK6 in U251 and T98G cells transfected with WT or hairpin A-deleted NEAT1. **J** ECAR was measured through the Glycolysis Stress in U251 cells transfected with WT or hairpin A-deleted NEAT1. **I** ECAR was measured through the Glycolysis Stress in T98G cells transfected with WT or hairpin A-deleted NEAT1. **K** Lactate concentration was assessed in U251 and T98G cells transfected with WT or hairpin A-deleted NEAT1. *p < 0.05, **p < 0.01. Error bars indicate mean ± SD of triple independent experiments

To further clarify the specific interaction region between NEAT1 and PGK1, a series of NEAT1 deletion mutants were constructed (Fig. 4A), and RNA pulldown assays were performed to examine the specific region binding to PGK1. The results showed that the 1–500 nt fragment of NEAT1 mediate its interaction with PGK1 (Fig. 4B). Moreover, the structural analysis using RNAfold software revealed a stable stem-loop (hairpin A) structure in the 1–500 nt range (Fig. 4C). To figure out whether the hairpin A is critical for association with PGK1, we generated site-directed mutagenesis of hairpin A followed by RNA pulldown and RIP assays. As shown in Fig. 4D, E, hairpin A mutation disrupted the interaction between NEAT1 and PGK1. Moreover, the hairpin A of NEAT1 was sufficient for its function in GBM cells (Fig. 4F–K).

**Fig. 5:**
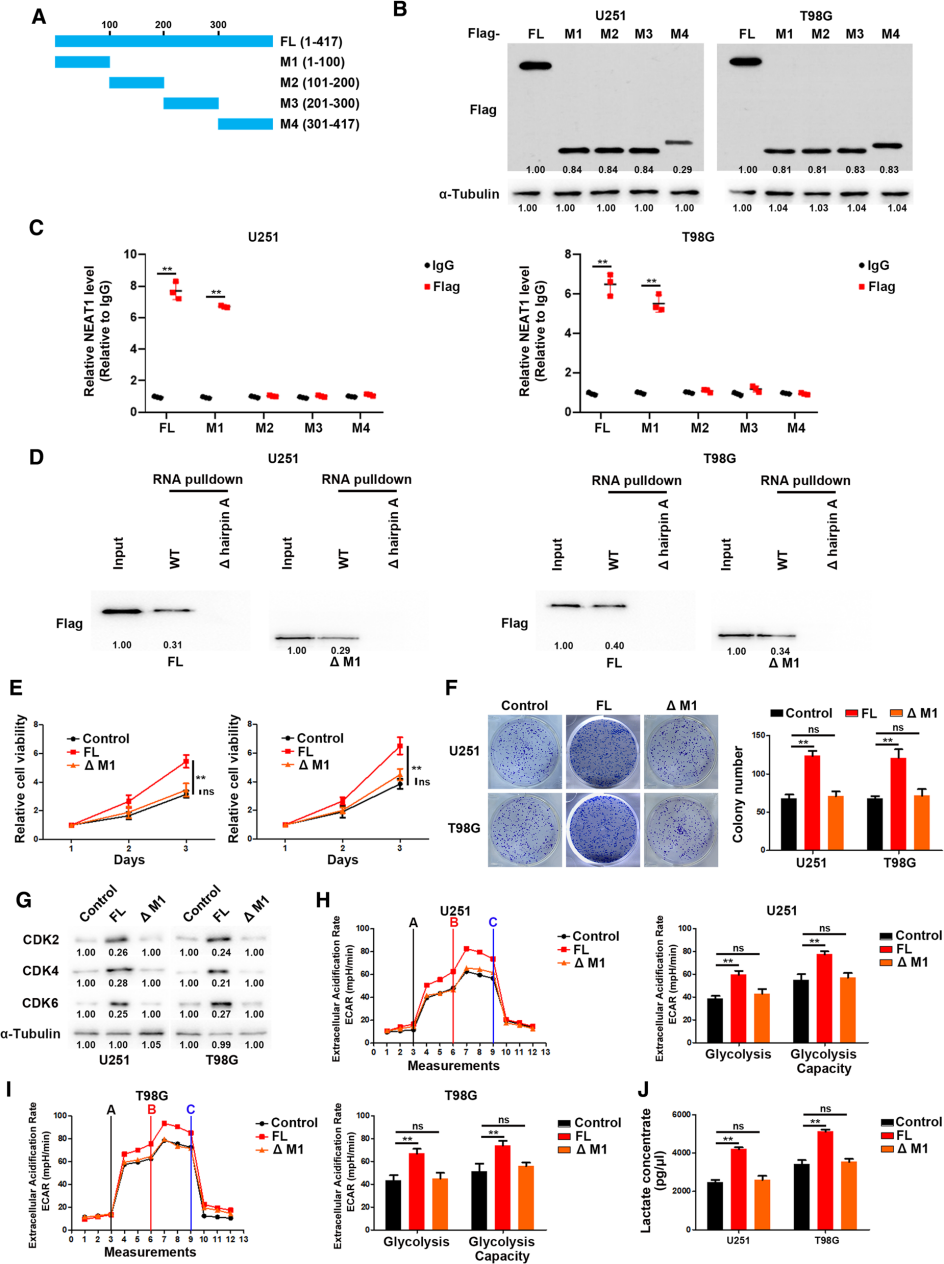
NEAT1 interacts with M1 residues of PGK1. **A** A schema of truncated PGK1. **B** Western blot analysis of U251 and T98G cells transfected with full length or truncated Flag-PGK1. **C** RIP assay showed the binding of NEAT1 to M1 residues of PGK1. **D** The deletion of M1 abolished the binding of PGK1 with NEAT1. **E** CCK-8 analysis of U251 and T98G cells transfected with full length or M1-deleted PGK1. **F** Colony formation assays of U251 and T98G cells transfected with full length or M1-deleted PGK1. **G** Western blot analysis of CDK2, CDK4, and CDK6 in U251 and T98G cells transfected with full length or M1-deleted PGK1. **H** ECAR was measured through the Glycolysis Stress in U251 cells transfected with full length or M1-deleted PGK1. **I** ECAR was measured through the Glycolysis Stress in T98G cells transfected with full length or M1-deleted PGK1. **J** Lactate concentration was assessed in U251 and T98G cells transfected with full length or M1-deleted PGK1. *p < 0.05, **p < 0.01. Error bars indicate mean ± SD of triple independent experiments

To map the minimal regions essential for the interaction between NEAT1 and PGK1, we generated several truncated mutants of Flag-PGK1 (Fig. 5A, B). Full-length and truncated PGK1 based RIP assays were performed, and the results showed that M1 residues of PGK1 are responsible for the interaction with NEAT1 (Fig. 5C). RNA pulldown experiments showed that M1 depletion abrogated the interaction between PGK1 and NEAT1, suggesting that NEAT1 mainly interacts with the M1 residues of PGK1 (Fig. 5D). In addition, we transfected glioma cells with full length or M1-depleted PGK1. As shown in Fig. 5E–J, M1 depletion significantly impaired the effect of PGK1 on proliferation and glycolysis of glioma cells.

Taken together, our results indicated that NEAT1 physically interacts with PGK1 to promotes cell proliferation and glycolysis.

### NEAT1/PGK1 promotes tumor progression in vivo

**Fig. 6:**
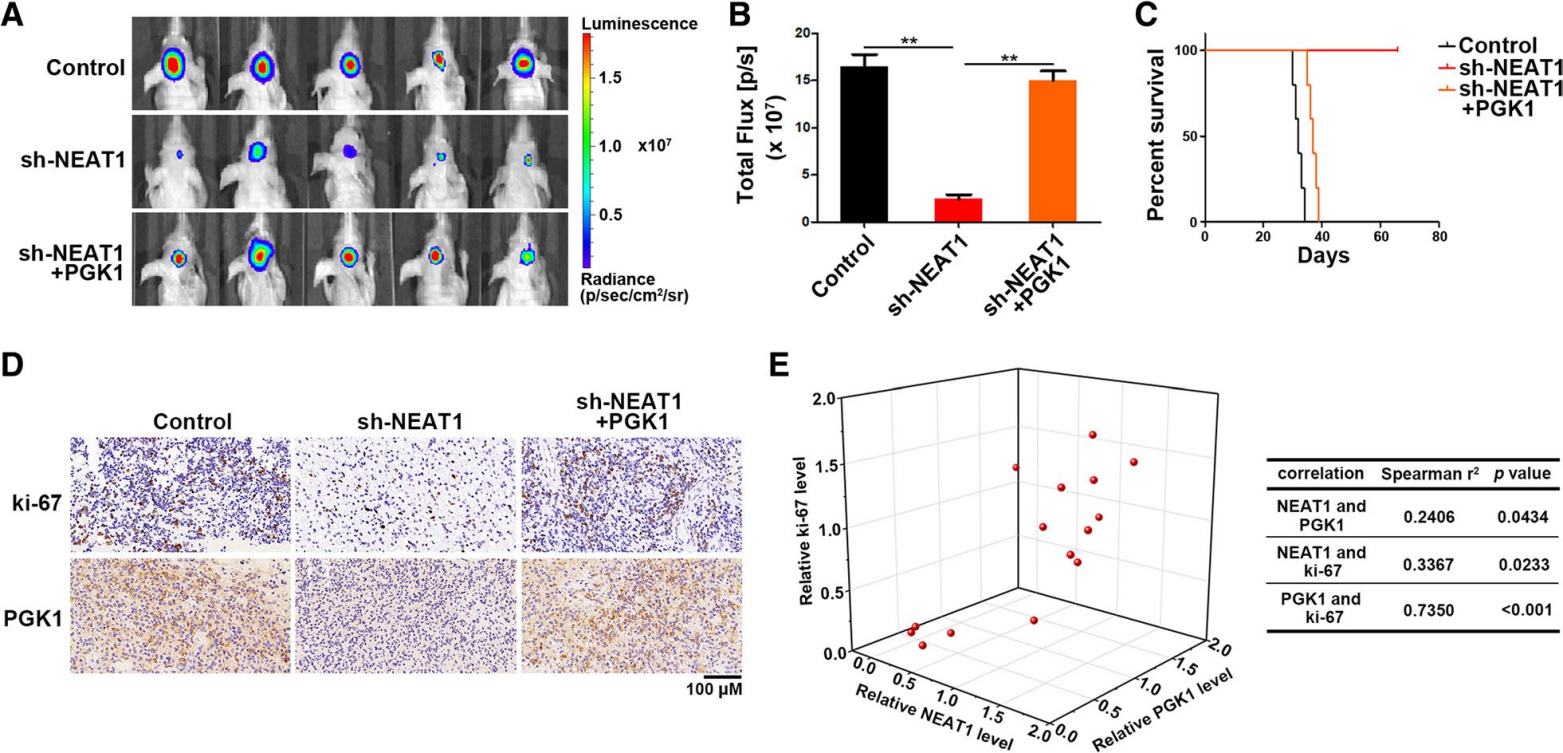
NEAT1/PGK1 promotes tumor progression in vivo. **A** Pseudocolor bioluminescence images of orthotopic tumors derived from U251 cells transfected with sh-NEAT1 or co-transfected with sh-NEAT1 and PGK1. **B** Bioluminescence was quantified in tumors from three groups. **C** Survival curves of mice from three groups. **D** Immunohistochemical analysis of ki-67 and PGK1 in tumors from three groups. **E** Three dimensional scatter plot of NEAT1, ki-67, and PGK1 in tumors from three groups. *p < 0.05, **p < 0.01. Error bars indicate mean ± SD

To investigate the regulation of NEAT1 on glioma cell proliferation and glycolysis of glioma in vivo, NEAT1-depleted or control U251 cells were intracranially injected into the brains of nude mice. As expect, NEAT1 knockdown significantly inhibited tumor growth (Fig. 6A, B). Moreover, NEAT1 knockdown prolonged the survival time of mice (Fig. 6C). In addition, PGK1 could reverse the inhibition of tumor growth caused by NEAT1 knockdown (Fig. 6A–C). IHC analysis indicated that NEAT1 knockdown decreased ki-67 and PGK1 expression, while PGK1 overexpression reversed ki-67 levels in tumors (Fig. 6D). At last, correlation analysis showed strong positive correlation between NEAT1, PGK1 and ki-67 in tumors (Fig. 6E). Taken together, NEAT1/PGK1 axis is indispensable for glioma growth.

### Characterization of NEAT1 as a biomarker for progression of glioma

**Fig. 7:**
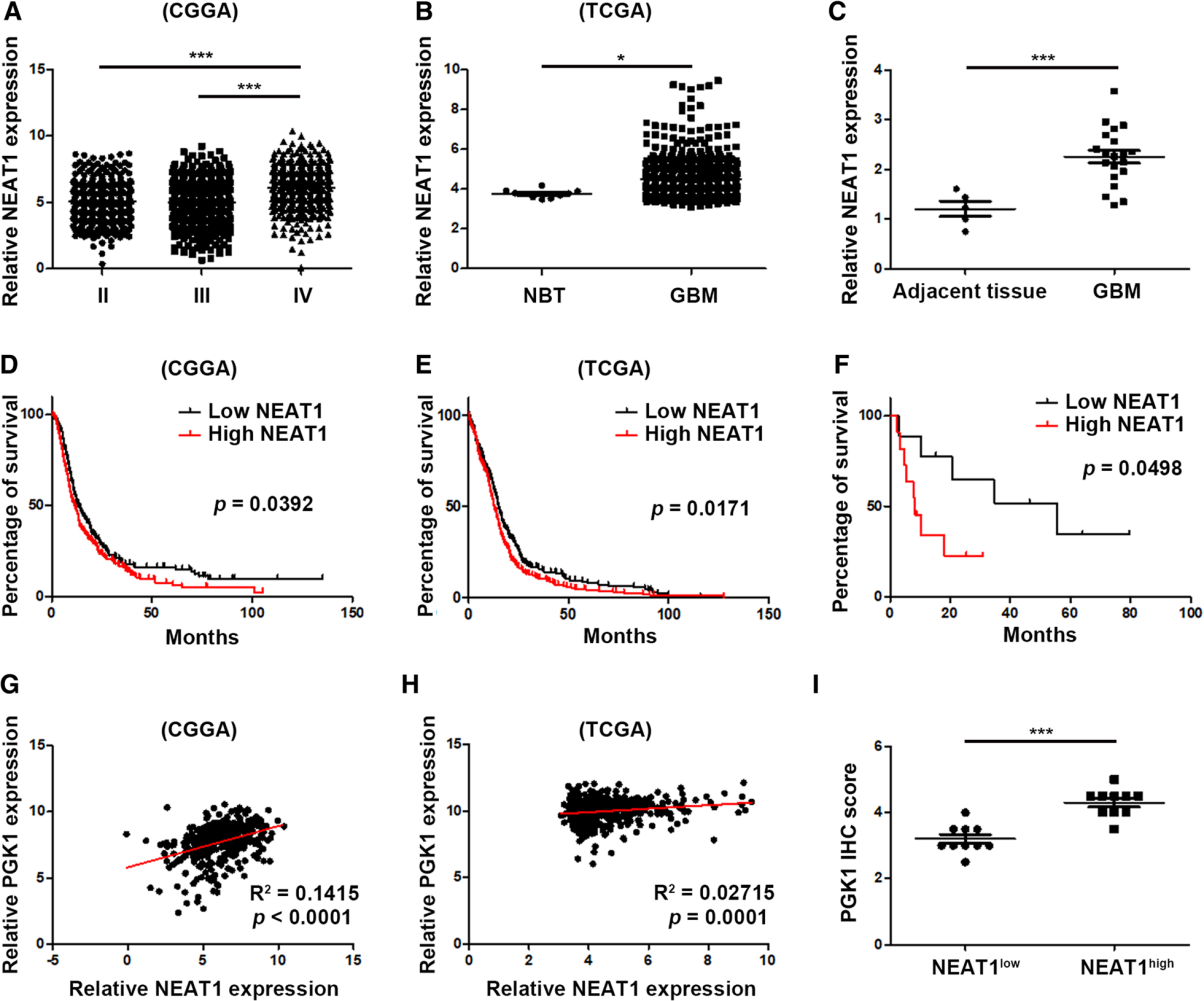
Characterization of NEAT1 as a biomarker for progression of glioma. **A** The CGGA database was used to analyze the expression of NEAT1 in different grade glioma samples. **B** The TCGA database was used to analyze the expression of NEAT1 in tumor adjacent tissues and GBM samples. **C** Twenty-five clinical samples were used to analyze the expression of NEAT1 in tumor adjacent tissues and GBM samples. **D** Overall survival (OS) of GBM patients with high or low NEAT1 expression from CGGA database. **E** Overall survival (OS) of GBM patients with high or low NEAT1 expression from TCGA database. **F** Overall survival (OS) of GBM patients with high or low NEAT1 expression from clinical samples. **G** The correlation between NEAT1 and PGK1 in GBM samples from CGGA database. **H** The correlation between NEAT1 and PGK1 in GBM samples from TCGA database. **I** PGK1 expression levels in GBM clinical samples with high or low NEAT1 levels. *p < 0.05, **p < 0.01. Error bars indicate mean ± SD

We analyzed the expression level of NEAT1 in glioma using TCGA and CGGA datasets. As shown in Fig. 7A, B, GBM tissues expressed higher level of NEAT1 than low grade glioma and normal brain tissues. In addition, we collected 5 normal brain tissues and 20 GBM samples. Concordantly, NEAT1 was significantly overexpressed in GBM samples (Fig. 7C). Moreover, NEAT1 was an independent prognostic predictor for poor survival of GBM patiens (Fig. 7D–F). In addition, NEAT1 expression was positively correlated with PGK1 in glioma (Fig. 7G–I). Together, these data suggest that NEAT1 serves as a predictive biomarker for poor outcome of GBM patients.

## Discussion

Recent studies have demonstrated that lncRNAs dramatically participate in many biological processes of tumors [17, 18]. Glioma is the most lethal tumor in central nervous system of adults. Despite of a great deal of researches on the treatment of glioma, the prognosis of glioma patients remains poor [1, 19]. LncRNAs have been considered to be involved in metabolism reprogramming, proliferation, invasion, radio- and chemo-resistance of glioma [20–24]. NEAT1 is a transcript of the multiple endocrine neoplasia type 1 (MEN1) gene located on human chromosome 11. It has been identified to be involved in various biological and pathological processes such as neurodegenerative diseases, viral infection [25–29]. At the same time, NEAT1 is one of the most investigated lncRNAs in glioma. Studies revealed that NEAT1 promotes glioma progression via activating several important signaling pathways, including mTOR and Wnt signaling. In nuclear, NEAT1 could interact with EZH2 to promotes H3K27 trimethylation levels, resulting the activation of Wnt signaling pathway [10]. In cytoplasm, NEAT1 functions as a ceRNA by sponging miR-185-5p to activate mTOR signaling [30]. Additionally, NEAT1 dysregulation was found to be involved in regulating the expression of multiple significant genes by recruiting or sequestering RNA-/DNA-binding proteins to or from promoters or target gene transcripts to influence gene transcription, splicing, RNA stability, or translation [31, 32]. However, the associated proteins of NEAT1 in cytoplasm have not been investigated. In this study, using RNA pulldown and mass spectrum analysis, we identified NEAT1 specifically interacted with PGK1 and blocked the degradation of PGK1. NEAT1 promotes glioma proliferation and glycolysis through regulating PGK1.

PGK1 is a pivotal ATP-generating enzyme in the glycolysis process, catalyzing the conversion of ADP and 1,3-BPG into ATP and 3-PG [33, 34]. Recent studies have shown that PGK1 is overexpressed and functions as an oncogene in numerous tumors, including glioma [35, 36]. The expression and kinase activity of PGK1 could be regulated by posttranslational modification. Ubiquitination is one of the most common and important modification of PGK1 [37]. PGK1 Ubiquitination level was regulated by lots of factors, including lncRNAs. In lung cancer, MetaLnc9 promoted migration and invasion of NSCLC cells. Mechanistic research indicated that MetaLnc9 specifically interacted with PGK1, which blocked PGK1 ubiquitination to activate AKT/mTOR signaling pathway [38]. In this study, we found for the first time that NEAT1 specifically interacted with PGK1 to block the ubiquitination of PGK1. Moreover, we precisely found that hairpin A of NEAT1 and M1 domain of PGK1 were the key connection points.

Clinically analysis using CGGA, TCGA datasets and our samples, we revealed that NEAT1 is overexpressed in GBM than low grade glioma and normal brain tissues. In addition, NEAT1 level was associated with poor prognosis of glioma patients. NEAT1 and PGK1 expression levels were positively correlated in glioma tissues, indicating NEAT1 and PGK1 are potential biomarkers for predicting glioma patients prognosis.

Collectively, our study found that NEAT1 enhances glioma cell proliferation and glycolysis in vitro and in vivo. Meanwhile, NEAT1 interacts with PGK1 increasing the stability of PGK1. Finally, we identified the role of NEAT1 as a potential biomarker and therapeutic target for glioma treatment.

## Data Availability

The data that support the findings of this study are available from the corresponding author upon reasonable request.

## Abbreviations

GBM: Glioblastoma
lncRNA: Long non-coding RNA
NEAT1: Nuclear paraspeckle assembly transcript 1
PGK1: Phosphoglycerate kinase 1
CDK2: Cyclin dependent kinase 2
CDK4: Cyclin dependent kinase 4
CDK6: Cyclin dependent kinase 6
